# Life expextancy of parents with Hereditary Haemorrhagic Telangiectasia

**DOI:** 10.1186/s13023-016-0427-x

**Published:** 2016-04-22

**Authors:** E. M. de Gussem, C. P. Edwards, A. E. Hosman, C. J. J. Westermann, R. J. Snijder, M. E. Faughnan, J. J. Mager

**Affiliations:** Department of Internal Medicine, University of Manitoba, Winnipeg, Canada; Department of Medicine, Division of Respirology, St Michael’s Hospital, University of Toronto, Toronto, Canada; Department of Pulmonology, St Antonius Hospital, Nieuwegein, The Netherlands; Li Ka Shing Knowledge Institute, St. Michael’s Hospital, Toronto, Canada

**Keywords:** Hereditary Haemorrhagic Telangiectasia, Genetics, Mortality, Pulmonary medicine, Vascular disease

## Abstract

**Background:**

Hereditary Haemorrhagic Telangiectasia (HHT) is an autosomal dominant disease associated with epistaxis, arteriovenous malformations and telangiectasias. Disease complications may result in premature death.

**Method:**

We investigated life-expectancies of parents of HHT patients compared with their non-HHT partners using self- or telephone-administered questionnaires sent to their children. Patients were extracted from the databases of 2 participating HHT Centres: the Toronto HHT Database (Toronto, Canada) and the St. Antonius Hospital HHT Database (Nieuwegein, The Netherlands).

**Results:**

Two hundred twenty five/407 (55 %) of respondents were included creating HHT- (*n* = 225) and control groups (*n* = 225) of equal size. Two hundred thirteen/225 (95 %) of the HHT group had not been screened for organ involvement of the disease prior to death. The life expectancy in parents with HHT was slightly lower compared to parents without (median age at death 73.3 years in patients versus 76.6 years in controls, p0.018). Parents with *ACVRL* 1 mutations had normal life expectancies, whereas parents with *Endoglin* mutations died 7.1 years earlier than controls (*p* = 0.024). Women with *Endoglin* mutations lived a median of 9.3 years shorter than those without (*p* = 0.04). Seven/123 (5 %) of deaths were HHT related with a median age at death of 61.5 years (IQ range 54.4–67.7 years).

**Conclusion:**

Our study showed that the life expectancy of largely unscreened HHT patients was lower than people without HHT. Female patients with *Endoglin* mutations were most strikingly at risk of premature death from complications. These results emphasize the importance of referring patients with HHT for screening of organ involvement and timely intervention to prevent complications.

## Background

Hereditary Haemorrhagic Telangiectasia (HHT) is an autosomal dominant disease with a prevalence of approximately 1/5000 [1]. HHT is diagnosed genetically, or clinically according to the Curaçao criteria [2]. Several disease causing mutations are known but most frequently patients are affected by mutations in the *Endoglin* (*ENG*; MIM 131195) gene (HHT-I) [3] or the *activin A receptor type II-like 1 (ACVRL1*; MIM 601284) gene (HHT-II) [4]. The Curaçao criteria are based on four clinical features: (i) the presence of spontaneous, recurrent epistaxis, (ii) mucocutaneous telangiectasia, (iii) visceral arteriovenous malformations (AVMs) and (iv) a first degree relative with HHT. Patients are diagnosed with HHT if they fulfil three out of four criteria. Two criteria results in a “possible HHT” diagnosis.

Telangiectasia can manifest on oral mucosa, nasal mucosa or on the face or fingers. Visceral AVMs mainly occur in the lungs (pulmonary AVM), brain (cerebral VM), gastro-intestinal tract and liver (hepatic VM) [5–7]. In patients with (possible) HHT, screening for organ involvement is recommended to prevent complications. These include intrapleural or intrabronchial haemorrhage from pulmonary AVMs or—more often—paradoxical (sterile or septic) emboli which can lead to a stroke or brain abscess [8–10]. Cerebral vascular malformations (VMs) can lead to haemorrhagic stroke, and liver VMs can lead to right congestive heart failure, portal hypertension, biliary necrosis or portosystemic encephalopathy [11]. Frequent recurrent epistaxis and gastro-intestinal telangiectasia, which mainly occur in the stomach and duodenum, can lead to iron deficiency anaemia [12].

To prevent complications and increase lifespan, patients should be referred to specialized HHT Centres for expert care and to screen for the presence of AVMs [2]. Previous studies in a small number of patients showed increased mortality in patients with HHT under 60 years of age [1, 13]. To study the life expectancy of patients who did not receive treatment for their (asymptomatic) AVMs, we evaluated the age of death of parents of the current population referred to our clinics. The aim was to determine whether life expectancy of unscreened parents of our cohorts with HHT differed from that of their non-HHT partner.

## Methods

### Population

The participating hospitals of this two-centre study are St. Michael’s Hospital in Toronto, Canada and St. Antonius Hospital in Nieuwegein, The Netherlands. Both hospitals are internationally recognized HHT Centres. Two thousand seven hundred and twenty-five patients screened for HHT were selected in June 2008 from the Toronto and St Antonius Hospital HHT Databases. The oldest generation known to the clinic with a confirmed genetic or clinical diagnosis of HHT, based on the Curaçao criteria, was included. Respondents’ admissions were excluded if a sibling had already returned a questionnaire on their parents or where HHT had been ruled out in both parents after screening in the clinic.

### Study protocol

The study protocol was approved by the research ethics boards of both participating hospitals (Medical Research Ethics Committees United). All selected patients received a letter with information on the study and a self-administered questionnaire. Patients were asked to return the questionnaire if both parents had deceased. If the HHT parent was still alive, patients were asked to forward the questionnaire to them with a request to complete it. If no answer was received within 4 weeks, patients were contacted by phone, by one of 2 well-trained interviewers (EG and CE), to answer the same questions.

The health status in terms of HHT of the parents of the participants was determined solely by the participants' response and was not objectified. However the participants used in this study had their diagnosis confirmed by the Curaçao criteria or genetic testing and genealogies often show which side of the family has HHT. Considering the hereditary nature of HHT the questions on which parent was affected by HHT allowed deduction of which parent was not affected by HHT. Subsequently, parents could then be assigned as HHT-patient or control. In case it was unclear which parent had HHT, because of a de novo mutation or when the HHT parent showed very little symptoms or in case of unclear genealogy, the participant was excluded.

By using the parent not affected by HHT as control, we assume the socioeconomic status of one set of parents is comparable, making the control group and HHT-group equal in socioeconomic status. The questionnaire queried the mortal status of both parents and, if applicable, the cause of death. Parents with HHT were divided in three groups: death definitely HHT related, possibly HHT related and not HHT related. Death secondary to pulmonary disease was categorized as possibly HHT related unless caused by pneumonia, emphysema, pulmonary embolism or tuberculosis; in these cases they were categorized as not HHT related deaths. Death secondary to an infection could be secondary to a paradoxical embolus leading to an abscess or sepsis and was categorized as possibly HHT related unless influenza or meningitis were the underlying causes; in these cases, they were categorized as not HHT related. Stroke was categorized as possibly HHT related. If death was reported secondary to cardiac disease it was categorized as possibly HHT related unless caused by a cardiac arrest, ruling it as not HHT related. To best interpret the results, the “possibly HHT related” group was then excluded from any further statistical analysis. The P-value was derived from cox-regression based on comparison of each of the two subgroups with controls.

Furthermore, the questionnaire queried HHT related symptoms in the HHT parent and asked whether this parent had received treatment for these symptoms or had been screened for HHT.

### Statistical analysis

Data was analysed using IBM SPSS statistics 22 and Microsoft Office Excel 2010. Survival data was analysed using Cox regression and Kaplan-Meijer curves. Given the inherent limitations of survey methodology, to assess whether survival of the control group was realistic, calculations from survival data reported for controls in the current survey were compared to the survival data of Dutch people in the national Dutch database (CBS statLine) and Canadian people in the national Canadian database (CANSIM).

## Results

Of the 570 questionnaires sent out, 407 (71 %) were completed, 9 (2 %) were returned because of an incorrect address and 154 (27 %) patients did not return the questionnaire or respond to phone calls. Of the respondents, 182 (45 %) were excluded for the following reasons: incomplete response (24), parent without HHT was still alive (73), neither parent was diagnosed with HHT or had symptoms (the respondent was then assumed to have a *de novo* mutation), parent had a low grade phenotype or one of the parents was not the genetic parent (52), or there were siblings among the respondents (to avoid dataset duplicates) (33).

In total 225 (55 %) respondents were included, 85/225 were from St. Michael’s Hospital and 140/225 were from St. Antonius Hospital, providing information on 550 parents; 225 HHT parents, and 225 controls. Of 157 (69.8 %) HHT parents, the gene mutation causing HHT was known. In the control group, males had shorter lifespans (median age 74.0 years, IQ range 65.7–80.9) than females (median age 80.1 years, IQ range 72.2–86.8), with a hazard ratio of 1.684 (95 % CI 1.284–2.208, *p* < 0.001) (Table 1).

**Table 1:** Comparing age at death (years) of parents with HHT (HHT group) and parents without HHT (control group), subdivided by sex and mutation

	HHT group				Control group			
	n^o^	Median age (years)	IQ range 25 % (years)	IQ range 75 % (years)	n^o^	Median age (years)	IQ range 25 % (years)	IQ range 75 % (years)
Total population	225	73.3	63.6	81.7	225	76.6	68.5	83.2
*Endoglin*	83	69.5	59.3	81.4				
*ACVRL1*	74	76.0	67.1	82.9				
Male	98	70.9	62.1	79.7	127	74.0	65.7	80.9
*Endoglin*	35	68.5	57.4	77.8				
*ACVRL1*	31	73.4	66.1	81.2				
Female	127	75.7	64.0	82.5	98	80.1	72.2	86.8
*Endoglin*	48	70.8	61.2	82.4				
*ACVRL1*	43	77.0	68.4	83.1				

This was as expected from the general Dutch and Canadian population (CBS statLine, CANSIM). The HHT group showed similar trends with respect to sex, but the difference was not statistically significant (hazard ratio 1.255, 95%CI 0.962–1.637, *p* = 0.096).

**Fig. 1:**
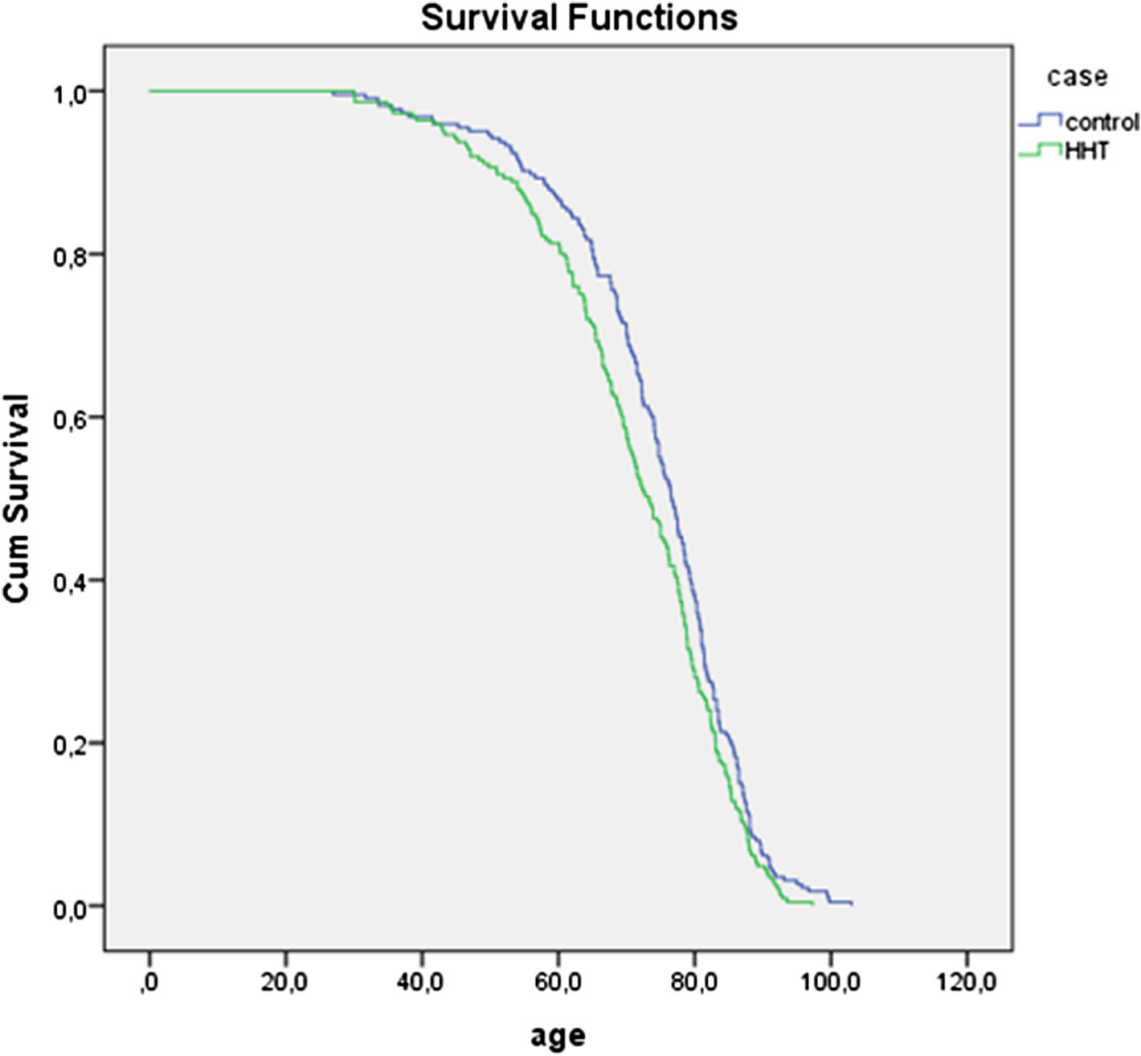
Kaplan-Meijer curve of HHT group versus control group. Log Rank: *p* = 0.018

**Fig. 2:**
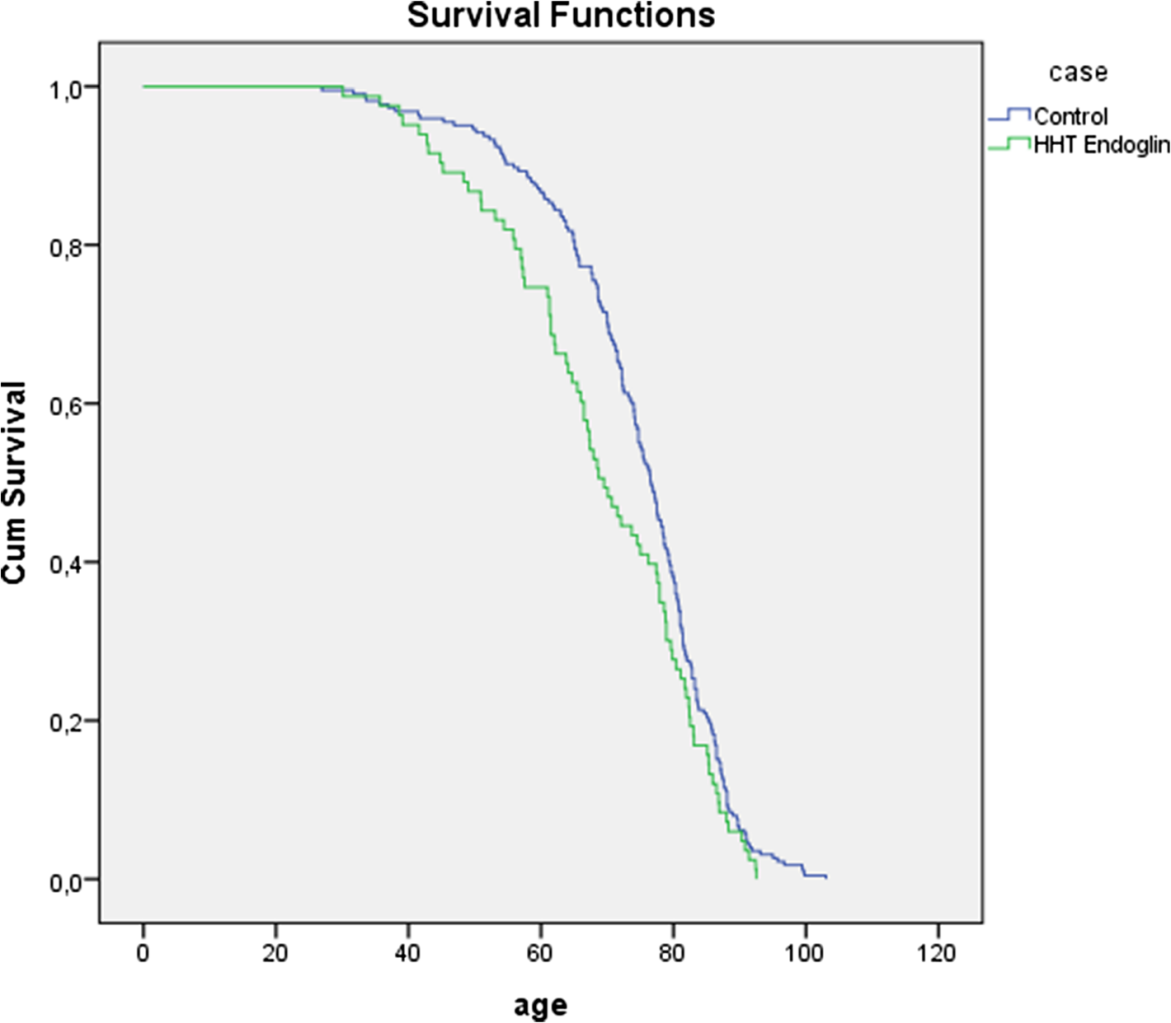
Kaplan-Meijer curve of unscreened *Endoglin* patients vs controls. Log Rank: *p* = 0.024

**Fig. 3:**
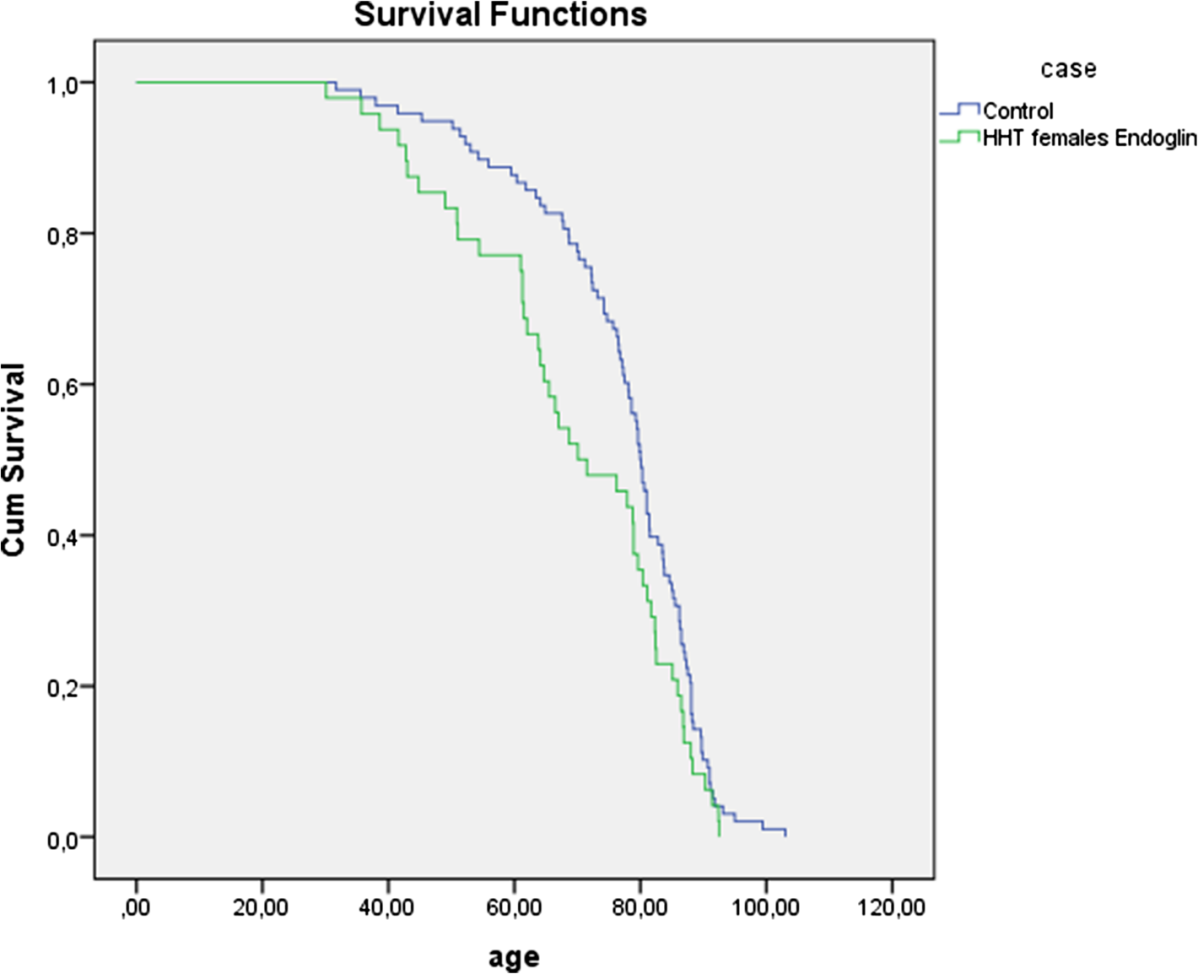
Kaplan-Meijer curve of unscreened female *Endoglin* patients vs female controls. Log Rank: *p* = 0.038

**Fig. 4:**
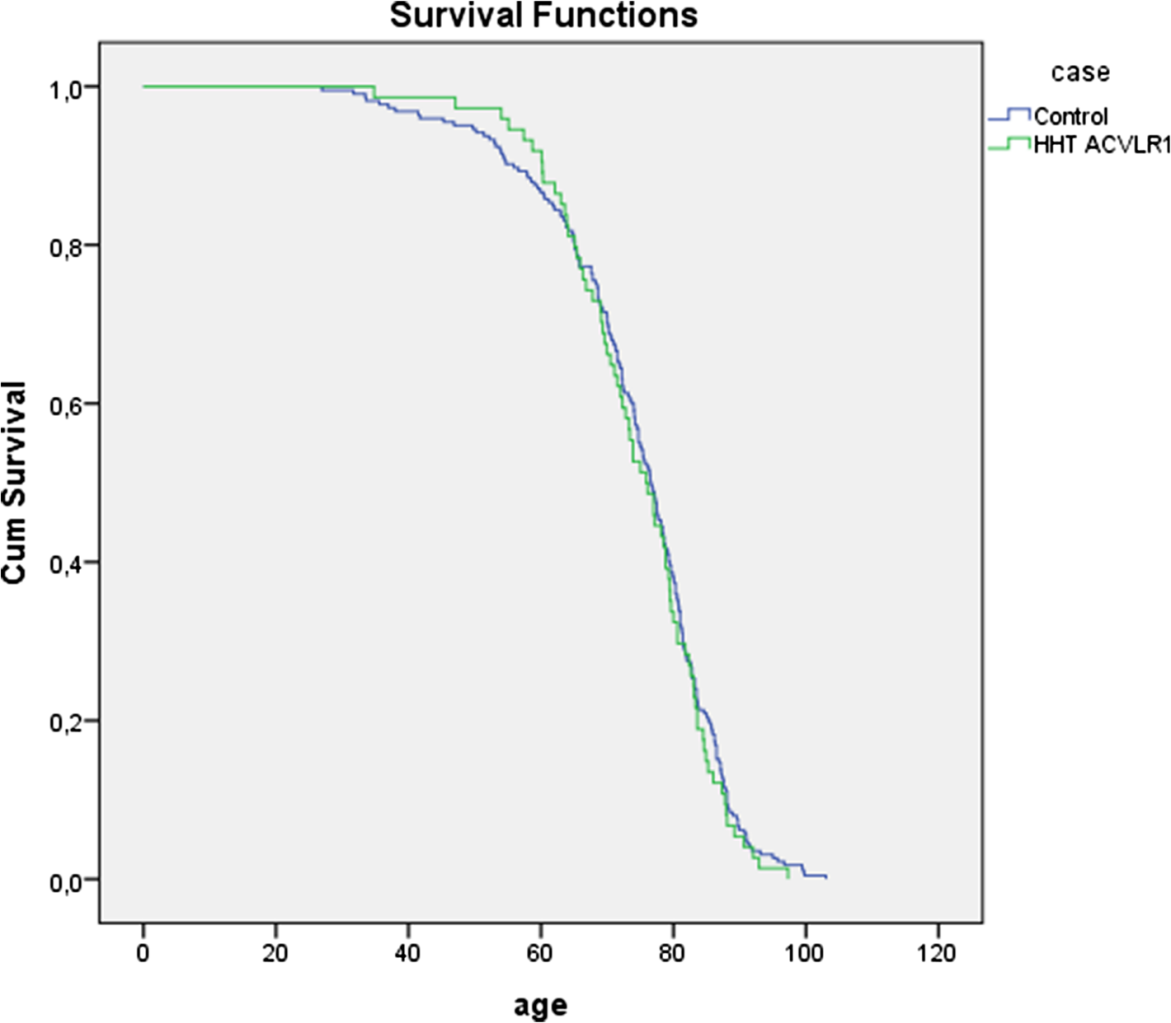
Kaplan-Meijer curve of unscreened *ACVLR1* patients vs controls. Log Rank: *p* = 0.476

Parents with HHT had a significantly shorter lifespan than parents without HHT. With a median age at death of 73.3 years (IQ range 63.3, 81.7) in the HHT group compared to a median age at death in controls of 76.6 years (IQ range 68.5, 83.2), HHT parents died 3.3 years earlier than controls (HR 1.252, 95 %CI 1.039–1.508, *p* = 0.018) (Fig. 1). Further analysis shows this difference could be attributed specifically to an *Endoglin* mutation. Parents with *Endoglin* mutations died significantly earlier than controls (HR 1.338, IQ range 1.038–1.723, *p* = 0.024) (Fig. 2). More strikingly, females with an *Endoglin* mutation lived a median of 9.3 years shorter than females without the mutation (HR 1.442, 95 % CI 1.017–2.043, *p* = 0.04) (Fig. 3). Males with an *Endoglin* mutation seemed to live 5.5 years shorter than non-HHT males, although this was not significant and may be because the data were underpowered (HR 1.361, IQ range 0.934–1.984, *p* = 0.109). By contrast, the lifespan of parents with *ACVRL1* mutations was not significantly different from those without (HR 1.101, 95 % CI 0.846–1.433, *p* = 0.476) (Fig. 4).

Parents with HHT who had died because of an HHT related complication had a lower median age at death compared to those who had died from no HHT related conditions (Table 2).

**Table 2:** Causes of death of parents with HHT. P-value derived from cox-regression based on comparison with controls

Cause of death HHT related	Yes	No
Number (%)	6 (5 %)	123 (95 %)
Median age (IQ range)	61.5	75.0
	(54.4–67.7)	(65.9–82.8)
Range	41.6–74.9	30.0–97.3
Causes of death (number)	Massive epistaxis (2)	Pulmonary disease (16)
	Pulmonary hemorrhage (2)	Cardiac disease (21)
	Brain abscess (3)	Infection (2)
		Abdominal aorta aneurysm (1)
		Malignancy (47)
		Bladder cyst (1)
		Dementia (2)
		Renal failure (2)
		Trauma (7)
		Unnatural (2)
		Old age (22)
P-value	0.002	0.343

Of the seven parents who died from complications of HHT, two had confirmed mutations in the *Endoglin* gene and one had a mutation in the *ACVRL1* gene. Four (57 %) of them were women.

Screening and treatment of HHT parents was limited. Only 5.3 % of parents with HHT were screened for the presence of organ VMs and 8.9 % and 1.3 % were treated for pulmonary AVMs and cerebral VMs respectively.

## Discussion

This study illustrates that in an essentially unscreened and untreated population of HHT patients who were the parents of cohorts in our databases, the median life expectancy is significantly lower than that of their non-HHT partner. This is in alignment with a recent study performed on 675, largely unscreened, HHT patients showing a similar 3 year reduction in life expectancy, mainly related to neurological and haemorrhagic complications [14]. More detailed analysis of our data showed that the decrease in life expectancy in the otherwise unspecified HHT cohort is largely attributable to reduced life expectancy in women with a mutation in the *Endoglin* gene. Life expectancy of individuals with *ACVRL 1* mutations did not seem to be affected.

Reduced life expectancy is more pronounced in female with an *Endoglin* mutation, for reasons that are mainly unclear although we can hypothesize. Possible reasons might include the nature of the *Endoglin* phenotype, which is associated with a higher prevalence of pulmonary AVMs and cerebral VMs compared to the *ACVRL1* mutations. Cerebral VMs occur in approximately 8–16 % of patients with *Endoglin* phenotype as opposed to 1–2 % of HHT-II patients with *ACVRL1* mutation [15–18]. Pulmonary AVMs occur much more frequently in patients with *Endoglin* mutations (62 %) compared to patients with *ACVRL1* mutations (10 %) and complications may be life threatening [19]; Major pulmonary AVM related complications include life-threatening hemorrhage, as well as cerebral abscess (8 %) and ischemic stroke (9 to 18 %) due to paradoxical emboli, which can be fatal or leave patients severely physically impaired [20, 21]. Fortunately, these complications can mostly be prevented through detection of pulmonary AVM by screening and treatment [22].

Even though women with HHT have a higher risk of life threatening pulmonary AVM-related complications during pregnancy and possibly labour [23–26] this did not appear to have a great impact on the study here: the overall life expectancy curves of women with *Endoglin* mutations only deviated in the 40–70 year range, and not during the fertile years (Fig. 2). However, this could be a result bias as women who died giving birth to their first child would not have had offspring to submit data for this study.

Seven parents whose death was definitely HHT related had a lower mean age at death compared to the two other groups. With present day standards of care, five of these deaths secondary to pulmonary AVMs, i.e. pulmonary haemorrhage and brain abscess, could have been prevented by screening and treatment in advance.

There are limitations to the methodology used in this study. The population included is biased, since it only includes adult respondents. Patients with HHT who died during childhood and in their twenties have been largely missed, although these numbers are likely very low [13, 27]. Due to stringent inclusion criteria, parents without prominent symptoms of HHT were also excluded, as they might not have had HHT and their responding child might have had a spontaneous mutation. However, these parents could have had a less evident phenotype or died before becoming clearly symptomatic and therefore might have been incorrectly excluded. Furthermore, studies on male HHT patients specifically were underpowered. Underpowering of these analyses can be explained by a smaller number of male patients. This may be due to a female predominance resulting from a greater inclination of Dutch female patients to be referred to an HHT Centre for clinical evaluation [16]. Furthermore, we excluded 73 participants of which the parent without HHT was still alive. Of 53/73 (73 %) participants, the deceased parent with HHT was male, this is consistent with a generally lower life expectancy for men compared to women. However, this led to a relatively smaller male HHT-group included in the study.

We studied life expectancy in a largely unscreened population in which only 5 % of the participants had been examined in a specialized clinic. The goal of current screening is to prevent HHT related complications and improve life expectancy in HHT patients. Future studies will show if morbidity and mortality improve if patients are screened for visceral AVMs and treated when necessary.

To improve life expectancy in patients with HHT or suspected HHT, we strongly recommend referral to an HHT Centre of Excellence for appropriate screening and possible treatment of AVMs. International HHT guidelines recommend screening for pulmonary AVMs in all people with HHT and also recommend referral to expert centres for management of the various aspects of HHT. Taking the hereditary nature of HHT in account, asymptomatic family members should also be referred for screening since clinical manifestations like epistaxis and telangiectasia increase with age and might not be evident initially. Asymptomatic AVMs can be present in the young, potentially leading to life threatening complications. Furthermore, increasing awareness of HHT is essential in order to improve patient care because despite greater awareness, HHT is still an under-diagnosed disease [28, 29].

## Conclusion

In conclusion, our study confirms the findings of recent literature that life expectancy in a largely unscreened population with HHT is worse than in their non-HHT partners [14], more specifically for patients with *Endoglin* mutations and especially women. Because patients with *ACVRL1* mutations have a normal life expectancy, the reduction in life expectancy in patients with an *Endoglin* mutation is probably related to complications of untreated pulmonary AVMs and cerebral VMs. We propose that life expectancy in HHT can be normal if patients are screened and both pulmonary AVMs and cerebral VMs are properly treated in a timely way. To prevent complications of HHT, referral of patients with (suspected) HHT to an HHT Centre of Excellence for screening, and if necessary treatment, is highly recommended.

## Abbreviations

HHT: Hereditary Haemorrhagic Telangiectasia
ACVRL1: activin A receptor type II-like 1
AVMs: arteriovenous malformations
VM: vascular malformations
HR: hazard ratio
CI: confidence interval
IQR: interquartile range

## References

[1] Kjeldsen AD, Vase P, Green A (1999). Hereditary haemorrhagic telangiectasia: a population-based study of prevalence and mortality in Danish patients. J Intern Med.

[2] Shovlin CL, Guttmacher AE, Buscarini E, Faughnan ME, Hyland RH, Westermann CJ, Kjeldsen AD, Plauchu H (2000). Diagnostic criteria for hereditary hemorrhagic telangiectasia (Rendu-Osler-Weber syndrome). Am J Med Genet.

[3] McDonald MT, Papenberg KA, Ghosh S, Glatfelter AA, Biesecker BB, Helmbold EA, Markel DS, Zolotor A, McKinnon WC, Vanderstoep JL (1994). A disease locus for hereditary haemorrhagic telangiectasia maps to chromosome 9q33-34. Nat Genet.

[4] Johnson DW, Berg JN, Baldwin MA, Gallione CJ, Marondel I, Yoon SJ, Stenzel TT, Speer M, Pericak-Vance MA, Diamond A, Guttmacher AE, Jackson CE, Attisano L, Kucherlapati R, Porteous ME, Marchuk DA (1996). Mutations in the activin receptor-like kinase 1 gene in hereditary haemorrhagic telangiectasia type 2. Nat Genet.

[5] Krings T, Kim H, Power S, Nelson J, Faughnan ME, Young WL, terBrugge KG, Brain Vascular Malformation Consortium HHT Investigator Group (2015). Neurovascular manifestations in hereditary hemorrhagic telangiectasia: imaging features and genotype-phenotype correlations. AJNR Am J Neuroradiol.

[6] Memeo M, Stabile Ianora AA, Scardapane A, Buonamico P, Sabba C, Angelelli G (2004). Hepatic involvement in hereditary hemorrhagic telangiectasia: CT findings. Abdom Imaging.

[7] Guttmacher AE, Marchuk DA, White RI (1995). Hereditary hemorrhagic telangiectasia. N Engl J Med.

[8] Woodall MN, McGettigan M, Figueroa R, Gossage JR, Alleyne CH (2014). Cerebral vascular malformations in hereditary hemorrhagic telangiectasia. J Neurosurg.

[9] Dupuis-Girod S, Giraud S, Decullier E, Lesca G, Cottin V, Faure F, Merrot O, Saurin JC, Cordier JF, Plauchu H (2007). Hemorrhagic hereditary telangiectasia (Rendu-Osler disease) and infectious diseases: an underestimated association. Clin Infect Dis.

[10] Mathis S, Dupuis-Girod S, Plauchu H, Giroud M, Barroso B, Ly KH, Ingrand P, Gilbert B, Godeneche G, Neau JP (2012). Cerebral abscesses in hereditary haemorrhagic telangiectasia: a clinical and microbiological evaluation. Clin Neurol Neurosurg.

[11] Garcia-Tsao G, Korzenik JR, Young L, Henderson KJ, Jain D, Byrd B, Pollak JS, White RI (2000). Liver disease in patients with hereditary hemorrhagic telangiectasia. N Engl J Med.

[12] Proctor DD, Henderson KJ, Dziura JD, Longacre AV, White RI (2005). Enteroscopic evaluation of the gastrointestinal tract in symptomatic patients with hereditary hemorrhagic telangiectasia. J Clin Gastroenterol.

[13] Sabba C, Pasculli G, Suppressa P, D'Ovidio F, Lenato GM, Resta F, Assennato G, Guanti G (2006). Life expectancy in patients with hereditary haemorrhagic telangiectasia. QJM.

[14] Donaldson JW, McKeever TM, Hall IP, Hubbard RB, Fogarty AW (2015). Complications and mortality in hereditary hemorrhagic telangiectasia: A population-based study. Neurology.

[15] Velthuis S, Vorselaars VM, van Gent MW, Westermann CJ, Snijder RJ, Mager JJ, Post MC (2013). Role of transthoracic contrast echocardiography in the clinical diagnosis of hereditary hemorrhagic telangiectasia. Chest.

[16] Letteboer TG, Mager JJ, Snijder RJ, Koeleman BP, Lindhout D, Ploos van Amstel JK, Westermann CJ (2006). Genotype-phenotype relationship in hereditary haemorrhagic telangiectasia. J Med Genet.

[17] Kjeldsen AD, Moller TR, Brusgaard K, Vase P, Andersen PE (2005). Clinical symptoms according to genotype amongst patients with hereditary haemorrhagic telangiectasia. J Intern Med.

[18] Bayrak-Toydemir P, McDonald J, Markewitz B, Lewin S, Miller F, Chou LS, Gedge F, Tang W, Coon H, Mao R (2006). Genotype-phenotype correlation in hereditary hemorrhagic telangiectasia: mutations and manifestations. Am J Med Genet A.

[19] van Gent MW, Post MC, Snijder RJ, Westermann CJ, Plokker HW, Mager JJ (2010). Real prevalence of pulmonary right-to-left shunt according to genotype in patients with hereditary hemorrhagic telangiectasia: a transthoracic contrast echocardiography study. Chest.

[20] Kjeldsen AD, Torring PM, Nissen H, Andersen PE (2014). Cerebral abscesses among Danish patients with hereditary haemorrhagic telangiectasia. Acta Neurol Scand.

[21] Shovlin CL, Jackson JE, Bamford KB, Jenkins IH, Benjamin AR, Ramadan H, Kulinskaya E (2008). Primary determinants of ischaemic stroke/brain abscess risks are independent of severity of pulmonary arteriovenous malformations in hereditary haemorrhagic telangiectasia. Thorax.

[22] Faughnan ME, Palda VA, Garcia-Tsao G, Geisthoff UW, McDonald J, Proctor DD, Spears J, Brown DH, Buscarini E, Chesnutt MS, Cottin V, Ganguly A, Gossage JR, Guttmacher AE, Hyland RH, Kennedy SJ, Korzenik J, Mager JJ, Ozanne AP, Piccirillo JF, Picus D, Plauchu H, Porteous ME, Pyeritz RE, Ross DA, Sabba C, Swanson K, Terry P, Wallace MC, Westermann CJ, White RI, Young LH, Zarrabeitia R, HHT Foundation International - Guidelines Working Group (2011). International guidelines for the diagnosis and management of hereditary haemorrhagic telangiectasia. J Med Genet.

[23] de Gussem EM, Lausman AY, Beder AJ, Edwards CP, Blanker MH, Terbrugge KG, Mager JJ, Faughnan ME (2014). Outcomes of pregnancy in women with hereditary hemorrhagic telangiectasia. Obstet Gynecol.

[24] Shovlin CL, Sodhi V, McCarthy A, Lasjaunias P, Jackson JE, Sheppard MN (2008). Estimates of maternal risks of pregnancy for women with hereditary haemorrhagic telangiectasia (Osler-Weber-Rendu syndrome): suggested approach for obstetric services. BJOG.

[25] Yeomans ER, Gilstrap LC (2005). Physiologic changes in pregnancy and their impact on critical care. Crit Care Med.

[26] Lapinsky SE, Kruczynski K, Slutsky AS (1995). Critical care in the pregnant patient. Am J Respir Crit Care Med.

[27] Giordano P, Lenato GM, Suppressa P, Lastella P, Dicuonzo F, Chiumarulo L, Sangerardi M, Piccarreta P, Valerio R, Scardapane A, Marano G, Resta N, Quaranta N, Sabba C (2013). Hereditary hemorrhagic telangiectasia: arteriovenous malformations in children. J Pediatr.

[28] Grosse SD, Boulet SL, Grant AM, Hulihan MM, Faughnan ME (2014). The use of US health insurance data for surveillance of rare disorders: hereditary hemorrhagic telangiectasia. Genet Med.

[29] Latino GA, Brown D, Glazier RH, Weyman JT, Faughnan ME (2014). Targeting under-diagnosis in hereditary hemorrhagic telangiectasia: a model approach for rare diseases?. Orphanet J Rare Dis.

